# Safety and efficacy of belimumab after B cell depletion therapy in systemic LUPUS erythematosus (BEAT-LUPUS) trial: statistical analysis plan

**DOI:** 10.1186/s13063-020-04391-2

**Published:** 2020-07-16

**Authors:** Patrick Muller, Kashfia Chowdhury, Caroline Gordon, Michael R. Ehrenstein, Caroline J. Doré

**Affiliations:** 1 Comprehensive Clinical Trials Unit, Institute of Clinical Trials and Methodology, University College London, London, UK; 2 Rheumatology Research Group, Institute of Inflammation and Ageing (IIA), University of Birmingham, Birmingham, UK; 3 Centre for Rheumatology, Division of Medicine, University College London, London, UK

**Keywords:** Statistical analysis plan, Systemic lupus erythematosus, Rituximab, Belimumab, Anti-dsDNA, Flare, British Isles Lupus Assessment Group, Causal mediation, Randomised controlled trial

## Abstract

**Background:**

There is limited evidence that rituximab, a B cell depletion therapy, is an effective treatment for systemic lupus erythematosus (SLE). Data on the mechanisms of B cell depletion in SLE indicate that the combination of rituximab and belimumab may be more effective than rituximab alone. The safety and efficacy of belimumab after B cell depletion therapy in systemic LUPUS erythematosus (BEAT-LUPUS) trial aims to determine whether belimumab is superior to placebo, when given 4–8 weeks after treatment with rituximab. This article describes the statistical analysis plan for this trial as an update to the published protocol. It is written prior to the end of patient follow-up, while the outcome of the trial is still unknown.

**Design and methods:**

BEAT-LUPUS is a randomised, double-blind, phase II trial of 52 weeks of belimumab versus placebo, initiated 4–8 weeks after rituximab treatment. The primary outcome is anti-dsDNA antibodies at 52 weeks post randomisation. Secondary outcomes include lupus flares and damage, adverse events, doses of concomitant medications, quality of life, and clinical biomarkers. We describe the trial’s clinical context, outcome measures, sample size calculation, and statistical modelling strategy, and the supportive analyses planned to evaluate for mediation of the treatment effect through changes in concomitant medication doses and bias from missing data.

**Discussion:**

The analysis will provide detailed information on the safety and effectiveness of belimumab. It will be implemented from July 2020 when patient follow-up and data collection is complete.

**Trial registration:**

ISRCTN: 47873003. Registered on 28 November 2016.

EudracT: 2015-005543-14. Registered on 19 November 2018.

## Trial summary and clinical context

### Clinical background and rationale

Systemic lupus erythematosus (SLE) is a chronic systemic autoimmune disease with a prevalence of 40–200 per 100,000 people, mainly affecting women of child-bearing age. There is a substantial morbidity and mortality associated with SLE, with standardised mortality ratios ranging from 2 to 5 [1]. There is also a lack of novel treatments for patients with severe SLE. Due to the lack of effective alternative therapies, many patients require high-dose glucocorticoid therapy which is associated with serious adverse effects including increased infections, cataracts, diabetes mellitus, and osteoporosis [2]. Both the disease itself and steroid exposure lead to increased rates of cardiovascular disease [3].

A key objective for treatment of severe SLE is disease remission induction and then prevention of “flare”; the worsening of lupus signs and symptoms in one or more systems of the body. It is expected that flares will be too rare in this phase II trial for any difference between treatment arms to be reliably detected, so anti-dsDNA antibody levels, which are a sensitive marker of immune system activity associated with flares [4], are the primary outcome instead. Clinical flares are a secondary outcome.

The biologic rituximab is currently the treatment of choice for refractory cases of SLE where other treatments have not succeeded, although no randomised controlled trials (RCTs) have demonstrated its effectiveness [5]. Previous studies have found that anti-dsDNA levels can increase in a proportion of patients treated with rituximab who then flare, leading us to hypothesise two effects of the medication: B cell depletion, which reduces flare risk; but also increasing levels of serum B cell activating factor/B lymphocyte stimulator (BAFF/BLyS) in certain patients, which increases the flare risk [6]. These opposing effects may explain the lack of significant efficacy found in the previous RCTs of rituximab.

Belimumab may be an effective addition to rituximab in this context, as it reduces BAFF levels. We therefore designed an early-phase clinical trial testing the safety and efficacy of rituximab followed by belimumab compared to rituximab alone [7]. Anti-dsDNA is a useful surrogate outcome to provide an early indication of the effectiveness of belimumab as an adjunct to rituximab, as it is correlated both with BAFF as well as flare activity.

Another marker of treatment effectiveness is a reduction in the patient’s steroid dose. Patients participating in BEAT-LUPUS will typically be on steroids or both steroids and immunosuppressants at the time of enrolment. During the trial, patients may take the steroid prednisolone and one immunosuppressant (either azathioprine, methotrexate, or mycophenolate). In usual care, their doctor will reduce their steroid dose if their condition improves and increase the dose if their condition deteriorates. Doctors participating in BEAT-LUPUS are asked to safely reduce their patient’s steroid dose if it is over 10 mg/day following administration of rituximab and belimumab/placebo. Differences between treatment arms in the extent to which steroid dose is actually reduced, and then maintained at a lower dose, will be partly determined by whether the treating clinician considers that this is safe and tolerable based on clinical symptoms following administration of belimumab or placebo.

### Trial objectives

The primary objective of BEAT-LUPUS is to compare anti-dsDNA levels 52 weeks after randomisation between a 52-week regime of either belimumab or placebo amongst patients treated with rituximab 4–8 weeks before randomisation. Lupus flares, incidence of adverse events, and changes in dosing of prednisolone are secondary outcomes. A supportive analysis will seek to examine whether any observed reductions in anti-dsDNA are mediated by changes in the prednisolone dose during follow-up.

## Study methods

### Design, randomisation, outcomes, and interim stopping rules

BEAT-LUPUS is a multicentre, phase II, randomised, double-blind, placebo-controlled clinical trial comparing safety and efficacy of a monthly regime of either belimumab or placebo commencing 4–8 weeks after B cell depletion therapy (rituximab). The total treatment period (on belimumab or placebo) is 52 weeks. There is an additional follow-up appointment at 56 weeks and a pregnancy check at 68 weeks. Full details of the interventions and study design are published in the trial protocol [7].

From March 2017 to March 2019, 52 patients were recruited and randomised 1:1 to receive either belimumab or placebo for 52 weeks after completing treatment with rituximab at one of 16 participating centres in the UK. Follow-up ended in April 2020, with the statistical analysis starting immediately afterwards.

Randomisation was done using minimisation incorporating a random element to ensure unpredictability in treatment allocations. Factors minimised include the CD19 count at randomisation (< 0.01 × 10^9^/l vs ≥ 0.01 × 10^9^/l) to account for variability in B cell depletion from rituximab, which would affect response; anti-dsDNA (positive or negative at first screen before rituximab); and whether patients have active renal disease at their first screen.

The primary analysis will utilise an analysis of covariance (ANCOVA) model, which will examine the treatment difference at 52 weeks and test for superiority. The measurement taken closest to 52-week follow up point will be used, with measurements taken up to 2 months before or after 52 weeks being eligible for inclusion in the analysis.

No formal interim analyses will be done. An Independent Data Monitoring Committee (IDMC) meet annually to review safety data, and may recommend stopping the trial if they judge the results are likely to convince a broad range of clinicians that one arm is clearly contraindicated.

### Sample size calculation

Sample size calculations were performed using STATA 13 [8]. The calculation assumed that anti-dsDNA binding antibody levels are log normally distributed, assumed that an ANCOVA model would be used to evaluate the difference in mean log anti-dsDNA between treatment arms at 52 weeks [9], and made additional adjustment for expected losses to follow-up.

The standard deviation of anti-dsDNA and the correlation structure were based on two sets of data: the study of 35 participants by Carter et al. [6]; and data provided by Professor David Isenberg of University College Hospital for 67 participants before and 6 months after B cell depletion therapy.

Based on the data presented in Table 1, the standard deviation of the final log anti-dsDNA measurements was assumed to be 1.7, and the correlation between baseline and final measurements was assumed to be 0.55. Twenty-two evaluable participants per group would be sufficient to detect a difference of 1.2 in log anti-dsDNA at 5% significance with 80% power. We assumed that 20% of participants would fail to attend the 12-month follow-up visit, so aimed to recruit 28 participants per group.

**Table 1:** Standard deviations and correlations for log anti-dsDNA

Dataset	Time point	Log mean	Log SD	Correlation
Isenberg (*n* = 67)	Baseline	6.036	1.393	
	Follow-up	5.189	1.500	0.612
Carter (*n* = 35)	Baseline	5.091	1.676	
	Follow-up	5.225	1.781	0.527

The study’s power to detect a difference of 1.2 in log anti-dsDNA is equivalent to being able to detect a difference of 232% between arms (equivalent to multiplying by exp.(1.2)). To put this in context, Carter et al. found that the log difference between participants who did and did not flare was 1.928, corresponding to a 588% increase in anti-dsDNA in those who flared [6].

## Statistical principles

Two-sided *p*-values and 95% confidence intervals will be reported for all statistical tests. There is one pre-specified primary analysis, which will use a *p*-value threshold of 5% to reject the null to ensure that the probability of a type I error does not exceed 5%. Log anti-dsDNA will be used to account for skewness in anti-dsDNA measurements.

Adherance to the protocol requires that the patient receives their randomised treatment for 52 weeks, does not exceed the pre-specified maximum doses of concomitant medication at enrolment, and also does not increase their doses of concomitant medications during follow up. Patients are encouraged to continue to provide follow-up measurements, even if they stop adhering to the protocol before 52 weeks.

The percentages of patients who fully adhere to the protocol and patients who do not adhere but do provide a 52-week measurement will be reported. The primary outcome analysis will be intention to treat; all patients who provide baseline and 52-week anti-dsDNA measurements will be included regardless of adherence to the protocol. Secondary analyses of the primary outcome will only include patients who adhered to the protocol.

## Trial population

The full eligibility criteria for enrolment into BEAT-LUPUS are listed in the published trial protocol [7] and Additional File 1: Inclusion and exclusion criteria.

Counts of patients screened but not enrolled in the trial and the reason for exclusion will be reported, and recruitment to the trial will be presented by centre and calendar month. The number of patients who withdraw or are unwilling to continue follow-up will be reported by the last follow-up visit attended and treatment arm. Reasons for patient withdrawals will be tabulated by treatment arm. The full throughput of patients from screening to analysis will be summarised in a CONSORT flowchart [10].

Baseline characteristics of patients in BEAT-LUPUS will be summarised by treatment arm (Additional File 1, Dummy Tables). Characteristics described will include screening anti-dsDNA, CD19 count, presence of renal disease, age, and sex. Characteristics at randomisation will also be reported: biomarker levels including anti-dsDNA, CD19 levels, and current doses of concomitant medications. Characteristics will be summarised using means and standard deviations for (approximately) normally distributed continuous variables, geometric means and 95% confidence intervals for (approximately) log normally distributed continuous variables, medians and interquartile ranges for non-normally distributed variables, and frequencies and percentages for categorical variables.

## Outcomes

### Primary and secondary outcomes

The primary outcome measure is log anti-dsDNA antibody levels at 52 weeks. Secondary outcomes are as follows:

1. Log anti-dsDNA antibody levels at 12 and 24 weeks
2. Proportion of participants with any adverse events and proportion with any serious adverse events
3. Proportion of participants with any infections
4. Proportion of participants with any severe flare (severe flare: a British Isles Lupus Assessment Group (BILAG-2004) A score due to items which are “new” or “worse” [11–13]; or, in the renal or haematological systems, an A score due to items that did not result in an A score last month) by 24 and 52 weeks, and time to severe flare
5. Proportion of participants with any severe flare or a moderate flare (moderate flare: two BILAG B scores due to items which are either “new” or “worse”; or, in the renal or haematological systems, B scores due to items that did not result in a B score last month) by 24 and 52 weeks, and time to severe or moderate flare
6. Proportion of participants with any severe flare, moderate flare, or mild flare (mild flare: a single BILAG B score due to items which are “new” or “worse”; or, for the renal or haematological systems, a B score due to items that did not result in a B score last month) by 24 and 52 weeks, and time to severe, moderate, or mild flare
7. Proportion of participants with any severe or moderate flare accompanied by an increase in concomitant lupus medication (glucocorticoids, mycophenolate, azathioprine, or methotrexate) by 24 and 52 weeks, and time to flare
8. Systemic Lupus Erythematosus Disease Activity Index 2000 (SLEDAI-2000) at 52 weeks [14]
9. Systemic Lupus International Collaborating Clinics/American College of Rheumatology (SLICC/ACR) damage index at 52 weeks [15]
10. Visual analogue scale (VAS) Subject Global Assessment of Disease Activity (SGADA) at 52 weeks
11. Complement C3 at 52 weeks
12. Immunoglobulin levels at 52 weeks
13. Cumulative steroid and immunosuppressant doses during treatment from randomisation to 52 weeks
14. Proportion of participants successfully reducing their steroid dose at the time of randomisation: decreasing their steroid dose by 50% without flaring; if below 10 mg/day at randomisation, reducing the steroid dose to 5 mg/day; or discontinue steroids with stable disease
15. Proportion of participants with a prednisolone dose ≤ 7.5 mg/day at both weeks 48 and 52
16. Lupus Quality of Life (Lupus QoL), Short Form 36 Health Survey (SF-36), and EQ-5D-5 L at 52 weeks [16–18]
17. Columbia Suicide Severity Rating Scale (C-SSRS) to assess suicide risk at 52 weeks [19]
18. Stanford HAQ 20-item Disability Scale (HAQ) at 52 weeks [20]

### Scoring and description of derived outcome measures

#### British Isles Lupus Assessment Group (BILAG-2004) index

The BILAG-2004 index questionnaire comprises 97 questions on lupus activity in the past 4 weeks compared to the previous 4 weeks, divided among 9 systems of the body [21]. Individual items are recorded either on a 0–4 ordinal scale from 0 = “not present” to 4 = “new”, or as real numbers (e.g. systolic blood pressure). An algorithm is then applied to determine an overall categorical score for each system depending on which items are present and how they are recorded; A = severe disease activity, B = moderate disease activity, C = mild disease, D = inactive disease but previously affected, and E = system never involved. Additional criteria are applied to identify A and B scores which are new manifestations of a flare of the disease. “Severe” flare occurs if there is at least one A score due to items which are “new” or “worse” on the BILAG questionnaire; or, in the renal and haematological systems, due to questionnaire items which last month did not result in an A score (i.e. which were less severe). A “moderate” flare occurs if at least two new B scores occur which are due to items which are “new” or “worse”, or, in the renal and haematological systems, due to items which last month did not result in a B score (i.e. were less severe). A “mild” flare occurs if there is only one B score which meets these conditions.

The subset of BILAG flares which are accompanied by an increase in one of the medications used to control the disease will also be evaluated. This allows evaluation of only those flares that were severe enough in the clinician’s judgement to modify the treatment regime.

#### The Systemic Lupus Erythematosus Disease Activity Index 2000 (SLEDAI-2000)

The SLEDAI Responder Index determines improvement in lupus activity based on 24 items in 9 organs in the previous 30 days [14]. The scores from the different systems are weighted in proportion to their hazard (i.e. central nervous system items are weighted as twice that of joint pain and kidney items) and combined into one final score from 0 to 105.

#### Patient global assessments of lupus activity on a 10-cm visual analogue scale (VAS)

This VAS is a BEAT-LUPUS-specific measure of disease activity developed for this trial. Patients are presented with a line labelled 0–10, and point to the number on the line which best matches their own assessment of lupus activity in terms of lupus-associated symptoms in the past 4 weeks (0 = not active at all, 10 = extremely active).

#### The Systemic Lupus International Collaborating Clinics/American College of Rheumatology (SLICC/ACR) damage Index for systemic lupus erythematosus

The SLICC/ACR Damage Index (SDI) provides a measure of accumulated damage in the body since the onset of lupus [22]. This summary score is based on damage across 12 different organ systems. For each system, a variety of different possible types of damage are listed, each scoring 1 point, and for some items a score or 2 is given if there has been more than one occurrence of the item, and for renal failure requiring renal replacement therapy a maximum score of 3 is given, and other renal items no longer score. The summary score for the whole body is the sum of all the individual scores.

#### LupusQoL

The LupusQol measure is a lupus-specific health-related quality of life measure [16]. It comprises 34 questions that each ask about effects of lupus on day-to-day physical and emotional health, body image, pain, planning, fatigue, intimate relationship, and burden to others. Patients answer each question on a scale from 1 = “all of the time” to 5 = “never”. Average scores for each domain are mapped to a 0–100 score. So long as 50% of data items for a domain are completed, a 0–100 score will be calculated, in line with guidance from the authors of the questionnaire [16]. The mean score across domains is then calculated as the average of the domain-specific scores.

#### The Short Form 36 Health Survey (SF-36)

The SF-36 is a survey of patient health in eight sections: vitality, physical functioning, bodily pain, general health perceptions, physical role functioning, emotional role function, social role functioning, and mental health [17]. Each section has a score that is a weighted sum of the questions in that section, directly transformed into a 0–100 score, with lower scores indicating more disability. Unanswered questions are excluded; the average for all items on the scale that the respondent answered is used instead. A standardised composite score of health is then generated from each of the eight scores.

#### EQ-5D-5 L

The EQ-5D-5 L assesses the current health state across five dimensions — mobility, self-care, usual activities, pain/discomfort, and anxiety/depression — with five levels (each scored 1–5, with higher scores indicating worse health state) [18]. EQ-5D dimension scores will be converted to index scores using UK population values. EQ-5D index scores range from − 1 = worse than death, and then 0 = worst to 1 = best health state. The EQ-5D additionally includes a visual analogue scale (EQ VAS), which allows patients to record their overall current health status on a scale ranging from 0 = worst to 100 = best health state.

If any dimension score is missing, the EQ-5D index score will be set to missing. If the entirety of one component of the questionnaire (dimension score or VAS) has not been completed, the associated component score will be set to missing. If the entire questionnaire has not been completed, both the EQ-5D index score and EQ-5D VAS at that visit will be set to missing.

#### Columbia Suicide Severity Rating Scale (C-SSRS)

The C-SSRS questionnaire provides summary measures of suicidal ideation and behaviour. These are strongly associated with the risk of an individual completing suicide [19]. The ideation and behaviour sections can be scored separately and also combined into one summary score [23]. Ideation is scored at each visit from 1 = “wish to be dead” to 5 = “active suicidal ideation with specific plan and intent”; behaviour is scored from 6 = “preparatory acts or behaviour” to 10 = “completed suicide”. Imputation of missing values is not done; if any data are missing for a domain, its score is not calculated.

#### The Stanford HAQ 20-item Disability Scale (HAQ)

This questionnaire summarises patient disability based on the extent of difficulty within eight domains; dressing and grooming, arising, eating, walking, hygiene, reach, grip, and activities [20]. The total score is the mean score of the eight category scores. If more than two of the categories are missing, the score is not calculated. If only one category is missing, the mean of the other seven category scores is used as the total score.

### Statistical analysis

The results of the analyses will be reported following the principle of the ICH E3 guidelines on the Structure and Content of Clinical Study Reports. All analyses will be performed using STATA 15 [8]. In addition, the primary analysis of the primary outcome, mediation, and other secondary analyses of the primary outcome will also be done, and results for the primary outcome will be presented by levels of the stratifying variables adjusted for in the primary analysis, as an exploratory subgroup analysis. For all analyses done using linear regression models, diagnostic checks will be done using residual plots and the data will be transformed and re-analysed if necessary. The results will be presented (Additional File 1, Dummy tables).

### Primary analysis of the primary outcome

A linear regression ANCOVA model will be fitted to evaluate the difference in 52-week anti-dsDNA between treatment arms, adjusting for CD19 count at randomisation (< 0.01 × 10^9^/l vs ≥ 0.01 × 10^9^/l), previous renal involvement (yes/no) at screening, log anti-dsDNA levels at screening, and also log anti-dsDNA levels measured at randomisation. Patients who provide these measurements will be included in the model and analysed according to their randomised treatment, regardless of treatment adherence. The model will be specified as follows, where *Y*_*i*,*j*_ is the anti-dsDNA of patient *j* at time *i*:

$$ \log \left({\mathrm{Y}}_{52,j}\right)={\beta}_0+{\beta}_1\left({\mathrm{treatment}}_j\right)+{\beta}_2\left(\mathrm{CD}{19}_j\right)+{\beta}_3\left(\log \left({\mathrm{Y}}_{0,j}\right)\right)+{\beta}_4\left({\mathrm{renal}}_j\right)+{\beta}_4\left(\log\ \left({\mathrm{screenDNA}}_j\right)\right)+{\varepsilon}_j $$

where treatment_*j*_ = 1 if belimumab and 0 if placebo, and *ε*_*ij*_ is a normal error distribution. The primary outcome will be estimated by exp(*β*_1_) as the difference in anti-dsDNA amongst patients randomised to belimumab compared to the placebo group at 52 weeks, expressed as a percentage of the average in the placebo group at 52 weeks.

### Supportive analyses of the primary outcome

#### Analysis of log anti-dsDNA at 12 and 24 weeks

The model structure used for the primary analysis will also be repeated with the outcome changed to log anti-dsDNA at 12 and 24 weeks to evaluate differences between treatment arms at these time points. These analyses will be done on the intention-to-treat basis, the same as the primary analysis.

#### Per-protocol repeated-measures analysis of anti-dsDNA at 52 weeks

Repeated-measures linear regression will be used to analyse the difference between arms in anti-dsDNA using the randomisation and all follow-up measurements in the same model. Measurements will be excluded after the point a patient stops adhering to the protocol; either the day after the patient fails to take their randomised treatment as scheduled, or from the second day after they increase the dose of one of the allowed concomitant medications (whichever comes first). This model will estimate the mechanistic effect of belimumab on anti-dsDNA.

In the model, Patient ID will be included as a random effect to account for correlation between measurements on the same patient at different points of follow-up. The model for anti-dsDNA at 52 weeks, where *y*_*ij*_ is the anti-dsDNA of patient *j* at time *i*, is:

$$ {y}_{ij}={\beta}_{0j}+{\beta}_1\left({\mathrm{time}}_i\right)+{\beta}_2\left({\mathrm{time}}_i\times {\mathrm{treatment}}_j\right)+{\beta}_3\left(\mathrm{CD}{19}_j\right)+{\beta}_4\left({\mathrm{renal}}_j\right) $$

where *β*_0*j*_ = *β*_0_ + *u*_0*j*_ + *ε*_*ij*_, *u*_0*j*_ ~ *N*(${\sigma}_{u0}^2$, 0), *ε*_*ij*_ ~ *N*(0, *σ*^2^), and treatment_*j*_ = 1 if belimumab and 0 if placebo.

The average treatment difference at 52 weeks will be estimated by *β*_2_ × 52. Log-transformation of anti-dsDNA or fractional polynomials for the effect of time will be considered if plots of residuals or likelihood ratio tests indicate that these will improve the model fit.

#### Mediation analysis of the effect of prednisolone on anti-dsDNA at 52 weeks

If material differences (*p* < 0.1) between treatment arms are found in the cumulative prednisolone dose between randomisation and 52 weeks, an exploratory causal mediation analysis will be done to evaluate the extent to which this may mediate any effect of allocation to belimumab on anti-dsDNA at 52 weeks [24]. The direct effect of belimumab (i.e. the effect of taking belimumab instead of placebo, had the cumulative steroid dose been the same in both conditions) and the average causal mediation effect (i.e. the effect of the cumulative steroid dose patients would have taken on belimumab instead of the dose they would have taken on placebo, had they actually taken belimumab in both conditions) will be estimated using the STATA *mediation* package [25].

#### Sensitivity analysis for informative loss to follow-up

If over 10% of patients fail to provide a 52-week anti-dsDNA measurement, a sensitivity analysis will be done using multiple imputation to evaluate whether the primary analysis and the repeated-measures per-protocol analysis are biased by missing data. Missing anti-dsDNA measurements will be imputed using all variables in the primary analysis model and data on concomitant medications, flares, and time to flare, all available anti-dsDNA measurements from other scheduled visits, and any anti-dsDNA measurements taken at point of flare/withdrawal. A number of imputation datasets sufficient to give a power reduction of < 1% compared to using *n* = 100 will be produced [26]; the analysis models will be run on each of these datasets; and estimates and confidence intervals will be combined using Rubin’s rules [27]. The concordance of results between the non-imputation (complete case) and imputation models will be assessed.

### Analysis of the secondary outcomes

The percentage of patients with the following characteristics will be compared between treatment arms using Fisher’s exact test:

I. BILAG severe flare
II. BILAG severe or moderate flare
III. BILAG severe, moderate, or mild flare
IV. BILAG severe or moderate flare which was accompanied by an increase in one or more concomitant medication
V. Any serious adverse event
VI. Any infection
VII. Any adverse events
VIII. Completed 52 weeks of follow-up
IX. Completed 52 weeks of treatment

For each of the SLEDAI, SLICC, VAS, C3, immunoglobulin levels, LupusQoL, SF-36, and EQ-5D-DL, assessments at 52 weeks will be compared between arms using linear regression models which include the stratifying variables and the value of the variable at screening (for the HAQ and SLICC) or randomisation (for all others). Time to disease flare will be visually displayed using Kaplan–Meier curves, and difference between arms in hazard of flare will be tested using Cox models that include the stratifying variables. For the BILAG flare scores, an ordinal logistic regression model will also be fitted to compare maximum disease flare severity experienced during follow-up (severe, moderate, mild, or no flare), also adjusted for the stratifying variables.

The following steroid dose summary measures will be compared between the arms:

i) Cumulative steroid dose from randomisation to 52 weeks using a two-sample *t* test
ii) Proportion of participants successfully reducing their steroid dose, using Fisher’s exact test
iii) Proportion of patients taking ≤ 7.5 mg of prednisolone at weeks 48 and 52

The following quantities taken from the C-SSRS will be compared between treatment arms:

i) Average C-SSRS score at 52 weeks
ii) Percentage of patients with a C-SSRS score which increased to > 5 at any point during follow-up

For the questionnaires completed at each follow-up visit (BILAG, VAS, and C-SSRS), if the questionnaire is not completed at one visit, then the result from the previous month will be carried forward for 1 month only (unless it is missing due to withdrawal/flare since the previous visit, in which case data captured at that point will be used).

## Discussion

This update to the published protocol describes the pre-specified statistical analysis plan for BEAT-LUPUS. By publishing it we aim to increase transparency of the data analysis, and demonstrate appropriate approaches for the challenges of: evaluating lupus activity; concomitant medications, which can vary between treatment arms post randomisation due to the trial treatment given and affect the primary outcome; and high expected loss to follow-up, a common feature of trials on severe SLE. By evaluating several measures of lupus activity, and using up-to-date statistical techniques to evaluate mediation of the treatment effect through changes in prednisolone dose and bias from missing data, we will return comprehensive and robust information on the safety and effectiveness of belimumab compared to placebo.

## Supplementary information

**Additional file 1.** Inclusion and exclusion criteria for BEAT-LUPUS, and dummy tables showing the planned format and contents of the tables for the final statistical report.

**Additional file 2.** BEAT-LUPUS CTU signed-off version of the SAP, containing further administrative details.

**Additional file 3.** Guidelines for the Content of Statistical Analysis Plans in Clinical Trials Checklist (as specified in Gamble et al, JAMA, 2017, and recommended by the EQUATOR Network).

## Data Availability

The protocol has previously been published [7]. Following completion of the trial analysis, the results will be published, and additional available data can be obtained by contacting the chief investigator (MRE). The study team retain exclusive use until publication of major outputs has been completed.

## Abbreviations

BAFF: B cell activating factor
BILAG: British Isles Lupus Assessment Group
C3: Complement component 3
C-SSRS: Columbia Suicide Severity Rating Scale
ICH: International Conference on Harmonisation
IDMC: Independent Data Monitoring Committee
SAP: Statistical Analysis Plan
SDI: SLICC/ACR Damage Index
SLE: Systemic lupus erythematosus
SLEDAI: Systemic Lupus Erythematosus Disease Activity Index
UCL: University College London
UCLH: University College Hospital
