# Supplementary material for: Safety and efficacy of belimumab after B cell depletion therapy in systemic LUPUS erythematosus (BEAT-LUPUS) trial: statistical analysis plan
Source: Trials. 2020 Jul 16;21:652. doi: 10.1186/s13063-020-04391-2 (PMC7364494; doi:10.1186/s13063-020-04391-2)
Supplement: Supplementary file 1 — Additional file 1. Inclusion and exclusion criteria for BEAT-LUPUS, and dummy tables showing the planned format and contents of the tables for the final statistical report. [file 13063_2020_4391_MOESM1_ESM.docx]

# BEAT-LUPUS SAP: Additional File

# 1. Inclusion and exclusion criteria

**Inclusion criteria:**

1. Aged between 18 and 75 years

2. Participants with 4 or more criteria for SLE according to the American College of Rheumatology (ACR) 1997 criteria or SLICC 2012 criteria or biopsy proven lupus nephritis with one additional supportive test on at least two occasions (positive ANA, anti-dsDNA antibodies or anti-Sm antibodies)

3. History of anti-dsDNA antibodies detectable at least once in the past 5 years prior to screening the participant on the study protocol (ELISA test is preferable for Anti dsDNA antibody testing).

4. Participants are due to be treated with the first infusion of this cycle of B cell depletion therapy (Rituximab) 4-8 weeks before randomisation (Day 0, see participant timeline). Previous use of Rituximab is allowed prior to this cycle.

5. No contraindications to the use of Belimumab.

6. Ability to provide informed consent

**Exclusion criteria:**

1. Severe “critical” SLE flare defined as BILAG A flare in CNS system or any SLE manifestation requiring more immunosuppression than allowed within the protocol in the physician's opinion

2. Pregnancy and/or Breast Feeding participants

3. At risk of pregnancy and unwilling to use an acceptable form of birth control contraception (see section 6.3.1.4)

4. Prior use of Belimumab, Atacicept or any biologic therapy (except Rituximab, but no other B cell depleting therapies)

5. Participation in any other interventional trial within the last 6 months

6. eGFR <30mls/min at screening

7. Active infections, including but not limited to:

i. Current or past infection with hepatitis B or C as defined by:

A. Hepatitis B surface antigen positive

B. Hepatitis B surface antibody positive and hepatitis B core antibody positive

C. Hepatitis C antibody positive

ii. Historically positive HIV test or test positive at screening for HIV

iii. Active TB.

8. Infection history:

i. Currently on any suppressive therapy for a chronic infection (such as tuberculosis, pneumocystis, cytomegalovirus, herpes simplex virus, herpes zoster and atypical mycobacteria)

ii. Hospitalization for treatment of infection within 60 days of Day 0

iii. Use of parenteral (IV or IM) antibiotics (antibacterials, antivirals, anti-fungals, or anti-parasitic agents) within 30 days of Day 0

9. Receipt of a live-attenuated vaccine within 3 months of Day 0 (see participant timeline)

10. In the investigator’s opinion, participants that are at high risk for infection (including but not limited to in dwelling catheter, dysphagia with aspiration, decubitus ulcer, history of prior aspiration pneumonia or recurrent severe urinary tract infection)

11. IgG levels below 4.0 g/L, IgA level < 10 mg/dL (IgG and IgA test must be performed no more than 10 days before study drug started for the second inclusion/exclusion criteria assessment at Day 0)

12. Primary immunodeficiency

13. History of malignant neoplasm within the last 5 years

14. History of cervical dysplasia CIN Grade III cervical high risk human papillomavirus or abnormal cervical cytology other than abnormal squamous cells of undetermined significance (ASCUS) within the past 3 years. The participant will be eligible after the condition has resolved (e.g., follow-up HPV test is negative or cervical abnormality has been effectively treated >1 year ago)

15. Severe, progressive, or uncontrolled renal, hepatic, haematological, gastrointestinal, pulmonary, cardiac, or neurological disease or, in the investigator’s opinion, any other concomitant medical condition or significant abnormal laboratory value that places the participant at risk by participating in this study with the exception of diseases or conditions related to active SLE.

16. Comorbidities, not lupus related currently requiring systemic corticosteroid therapy.

17. Evidence of serious suicide risk including any history of suicidal behaviour in the last 6 months and/or any suicidal ideation in the last 2 months or who in the investigator’s judgement, pose a significant risk.

18. History of an anaphylactic reaction to parenteral administration of contrast agents, human or murine proteins or monoclonal antibodies.

19. Current drug or alcohol abuse or dependence, or a history of drug or alcohol abuse or dependence within 364 days prior to Day 0

20. White blood cells (WBC) <1.5 x 109/L, Neutrophils <1 x 109/L measured up to 10 days before Day 0 (study drug commenced)

21. A history of major organ transplant or hematopoietic stem/cell/marrow transport or renal transplant.

# 2. Dummy Tables

**Table 1: Patient characteristics at screening (or at randomisation, if specified)**

| **Characteristic** |  | **Belimumab** | **Placebo** | **Total** |
| --- | --- | --- | --- | --- |
|  |  | n= | n= | n= |
| Age (years) | mean(sd) |  |  |  |
| Female | n(%) |  |  |  |
| Weight (kg) | mean(sd) |  |  |  |
| Active renal disease | n(%) |  |  |  |
| CD19≥0.01x10^9^/L | n(%) |  |  |  |
| *BILAG A or B* |  |  |  |  |
| Constitutional | n(%) |  |  |  |
| Mucocutaneous |  |  |  |  |
| Neuropsychiatric |  |  |  |  |
| Musculoskeletal |  |  |  |  |
| Cardiorespiratory |  |  |  |  |
| Gastrointestinal |  |  |  |  |
| Ophthalmic |  |  |  |  |
| Renal |  |  |  |  |
| Haematological |  |  |  |  |
| *SLEDAI 2000* | mean(sd) |  |  |  |
| Score ≥10 | n(%) |  |  |  |
| *Organ involvement* |  |  |  |  |
| CNS | n(%) |  |  |  |
| Serosal |  |  |  |  |
| Haematological |  |  |  |  |
| Constitutional |  |  |  |  |
| Immunological |  |  |  |  |
| Musculoskeletal |  |  |  |  |
| Dermal |  |  |  |  |
| Renal Vascular |  |  |  |  |
| *SLICC damage index* | mean(sd) |  |  |  |
| *Subjective GADA* | mean(sd) |  |  |  |
| *Lupus QoL* | mean(sd) |  |  |  |
| Physical Health |  |  |  |  |
| Emotional Health |  |  |  |  |
| Body Image |  |  |  |  |
| Pain |  |  |  |  |
| Planning |  |  |  |  |
| Fatigue |  |  |  |  |
| Intimate relationships |  |  |  |  |
| Burden to others |  |  |  |  |
| *SF36* |  |  |  |  |
| Vitality | mean(sd) |  |  |  |
| Physical functioning |  |  |  |  |
| Bodily pain |  |  |  |  |
| General health |  |  |  |  |
| Physical role |  |  |  |  |
| Emotional role |  |  |  |  |
| Social role |  |  |  |  |
| Mental health |  |  |  |  |
| *C-SSRS* |  |  |  |  |
| Ideation | mean(sd) |  |  |  |
| Behaviour |  |  |  |  |
| *HAQ* |  |  |  |  |
| Total | mean(sd) |  |  |  |
| Dressing & Grooming |  |  |  |  |
| Arising |  |  |  |  |
| Eating |  |  |  |  |
| Walking |  |  |  |  |
| Hygiene |  |  |  |  |
| Reach |  |  |  |  |
| Grip |  |  |  |  |
| Activities |  |  |  |  |
| *SDI* | mean(sd) |  |  |  |
| *EQ-5D-5L* | mean(sd) |  |  |  |
| *VAS* | mean(sd) |  |  |  |
| *Medications at randomisation* |  |  |  |  |
| Daily prednisolone use | Mean(sd) |  |  |  |
| ≥7.5mg/day | n(%) |  |  |  |
| Any immunosuppressant use | n(%) |  |  |  |
| Mycophenolate mofetil |  |  |  |  |
| Azathioprine |  |  |  |  |
| Methotrexate |  |  |  |  |
| Antimalarial use |  |  |  |  |
| *Biomarkers at randomisation* |  |  |  |  |
| Anti-dsDNA antibodies IU/ml | mean(sd) |  |  |  |
| C3 g/L |  |  |  |  |
| C4 g/L |  |  |  |  |
| IgG g/L |  |  |  |  |
| IgA g/L |  |  |  |  |
| IgM g/L |  |  |  |  |
| *B cell subsets at randomisation* |  |  |  |  |
| CD19 x10^9^/L | mean(sd) |  |  |  |

**Table 2: Primary outcome, disease flares and adverse events**

| **Outcome** |  | **Belimumab** | **Placebo** | **Effect^#^** | **p-value** |
| --- | --- | --- | --- | --- | --- |
|  |  | n= | n= | (95% CI) |  |
| **Protocol compliance & concomitant medications** |  |  |  |  |  |
| Completed 52 wks follow up | n(%) |  |  |  |  |
| Completed 52 wks treatment | n(%) |  |  |  |  |
| Cumulative steroid dose from randomisation to 52wks | mean(95%CI) |  |  |  |  |
| Proportion of patients successfully reducing steroid dose by 50% or to 5mg/day without flaring | n(%) |  |  |  |  |
| Proportion of patients taking <=7.5 mg/day prednisolone at both weeks 48 and 52 | n(%) |  |  |  |  |
| **Anti-dsDNA** |  |  |  |  |  |
| **Anti-dsDNA antibodies IU/ml at 52wks**** | Geometric mean (95%CI) |  |  |  |  |
| Anti-dsDNA antibodies IU/ml at 24wks | Geometric mean(95%CI) |  |  |  |  |
| Anti-dsDNA antibodies IU/ml at 12wks | Geometric mean(95%CI) |  |  |  |  |
| Per-protocol repeated measures analysis estimates of Anti-dsDNA at 52wks | mean(95%CI) |  |  |  |  |
| **Disease flares** |  |  |  |  |  |
| Proportion with severe flare by 52wks | n(%) |  |  |  |  |
| Proportion with severe flare by 24wks | n(%) |  |  |  |  |
| Time to severe flare, wks | median(95%CI) |  |  |  |  |
| Proportion with severe/moderate flare by 52wks | n(%) |  |  |  |  |
| Proportion with severe/moderate flare by 24wks | n(%) |  |  |  |  |
| Time to severe/moderate flare, wks | median(95%CI) |  |  |  |  |
| Proportion with severe/moderate/mild flare by 52wks | n(%) |  |  |  |  |
| Proportion with severe/moderate/mild flare by 24wks | n(%) |  |  |  |  |
| Time to severe/moderate/mild flare, wks | median(95%CI) |  |  |  |  |
| Proportion with severe or moderate flare followed by increase in a concomitant medication by 52wks | n(%) |  |  |  |  |
| Proportion with severe or moderate flare followed by increase in a concomitant medication by 24wks | n(%) |  |  |  |  |
| Time to severe or moderate flare followed by increase in a concomitant medication | median(95%CI) |  |  |  |  |
| Odds ratio for having flare of greater severity (severe, moderate, mild, compared to no flare) from ordered logit model |  |  |  |  |  |
| **Adverse events** |  |  |  |  |  |
| Proportion with any SAEs by 52wks | n(%) |  |  |  |  |
| Proportion with any infections by 52wks | n(%) |  |  |  |  |
| Proportion with any adverse events by 52wks | n(%) |  |  |  |  |
| ^#^ Unless stated otherwise the intention to treat population is used, and effect estimates for continuous variables are from ANCOVA linear regression models which adjust for renal activity, anti-dsDNA at screening, and CD19 count and anti-dsDNA at randomisation. Those covariates are also adjusted for in the Cox regression models for time to flare, and ordered logit model for odds ratio for flare of greater severity. For anti-dsDNA only, the log(anti-dsDNA) is modelled and the model effect estimate is the percentage difference in anti-dsDNA between arms. For the per-protocol repeated measures analysis, the effect estimate is the model-based estimate of difference in log anti-dsDNA at 52 weeks. Fisher’s exact is used for comparison of proportions. | | | | | |
|  |  |  |  |  |  |
|  |  |  |  |  |  |
| ** Primary analysis of primary outcome | | | | | |

**Table 4: Secondary outcomes, disease activity, quality of life, and biomarkers at 52 weeks**

| **Outcome** |  | **Belimumab** | **Placebo** | **Effect^#^** | **p-value** |
| --- | --- | --- | --- | --- | --- |
|  |  | n= | n= | (95% CI) |  |
| SLEDAI 2000 score at 52wks | mean(95%CI) |  |  |  |  |
| SLICC Damage Index at 52 wks | mean(95%CI) |  |  |  |  |
| **Patient-reported outcomes** |  |  |  |  |  |
| Subjective GADA at 52wks | mean(95%CI) |  |  |  |  |
| *HAQ* |  |  |  |  |  |
| Total | mean(sd) |  |  |  |  |
| Dressing & Grooming |  |  |  |  |  |
| Arising |  |  |  |  |  |
| Eating |  |  |  |  |  |
| Walking |  |  |  |  |  |
| Hygiene |  |  |  |  |  |
| Reach |  |  |  |  |  |
| Grip |  |  |  |  |  |
| Activities |  |  |  |  |  |
| *Lupus QoL at 52wks* |  |  |  |  |  |
| Total  Physical Health | mean(sd) |  |  |  |  |
| Emotional Health |  |  |  |  |  |
| Body Image |  |  |  |  |  |
| Pain |  |  |  |  |  |
| Planning |  |  |  |  |  |
| Fatigue |  |  |  |  |  |
| Intimate relationships |  |  |  |  |  |
| Burden to others |  |  |  |  |  |
|  |  |  |  |  |  |
|  |  |  |  |  |  |
| *SF36* |  |  |  |  |  |
| Vitality | mean(sd) |  |  |  |  |
| Physical functioning |  |  |  |  |  |
| Bodily pain |  |  |  |  |  |
| General health |  |  |  |  |  |
| Physical role |  |  |  |  |  |
| Emotional role |  |  |  |  |  |
| Social role |  |  |  |  |  |
| Mental health |  |  |  |  |  |
|  |  |  |  |  |  |
| Average EQ-5D-5L from randomisation to 52wks | mean(95%CI) |  |  |  |  |
| C-SSRS at 52wks | mean(95%CI) |  |  |  |  |
| C-SSRS increase to >5 at any follow up visit | n(%) |  |  |  |  |
| **Biomarkers** |  |  |  |  |  |
| C3 at 52wks g/L | mean(95%CI) |  |  |  |  |
| IgG at 52wks g/L | mean(95%CI) |  |  |  |  |
| IgA at 52wks g/L | mean(95%CI) |  |  |  |  |
| IgM at 52wks g/l | mean(95%CI) |  |  |  |  |
| CD19 at 52wks x10^9^/L | mean(95%CI) |  |  |  |  |
| ^#^ Unless stated otherwise the intention to treat population is used, and effect estimates for continuous variables are from ANCOVA linear regression models which adjust for renal activity, anti-dsDNA, and CD19 count at randomisation. Fisher’s exact is used for comparison of proportions. | | | | | |
