# Supplementary material for: Safety and efficacy of belimumab after B cell depletion therapy in systemic LUPUS erythematosus (BEAT-LUPUS) trial: statistical analysis plan
Source: Trials. 2020 Jul 16;21:652. doi: 10.1186/s13063-020-04391-2 (PMC7364494; doi:10.1186/s13063-020-04391-2)
Supplement: Supplementary file 2 — Additional file 2. BEAT-LUPUS CTU signed-off version of the SAP, containing further administrative details. [file 13063_2020_4391_MOESM2_ESM.pdf]

BEAT Lupus

Safety and efficacy of Belimumab after B cell depletion therapy in systemic LUPUS erythematosus – BEAT LUPUS

**UCL CTU ID:**	**CTU/2013/096**
**UCL SPONSOR ID:**	**14/0665**
**Protocol version:**	**6.0 (15th July 2019)**
**International Standard RCT Number (ISRCTN):**	**47873003**
**European Clinical Trials Database Number (EudracT):**	**2015-005543-14**
**MHRA Clinical Trials Authorisation Number (CTA):**	**20363/0364/001-0001**
**Research Ethics Committee Number:**	**16/LO/1024**

Statistical Analysis Plan (SAP)

Version 1.1

**Approved by:**	**Signature**	**Date**
Professor Michael Ehrenstein<br>Chief Investigator	M. Ehrenstein	27 April 2020
Professor Caroline Doré<br>Professor of Clinical Trials and Statistics, CCTU	Caroline Doré	27 April 2020

Prepared by:

Patrick Muller
Trial Statistician
Email: p.muller@ucl.ac.uk
Tel: 020 3108 7320

Table of contents

1 ABBREVIATIONS AND GLOSSARY 2

2 TRIAL SUMMARY & CLINICAL CONTEXT 3

2.1 Clinical background and rationale 3
2.2 Trial objectives 4

3 STUDY METHODS 4

3.1 Design, randomisation, outcomes, and interim stopping rules 4
3.2 Sample size calculation 4

4 STATISTICAL PRINCIPLES 5

5 TRIAL POPULATION 6

Figure 1. Participant Timeline 7

6 TRIAL OUTCOMES 8

6.1 Primary and secondary outcomes 8
6.2 Scoring and description of derived outcome measures 9

7 STATISTICAL ANALYSIS 11

7.1 Primary analysis of the primary outcome 11
7.2 Supportive analyses of the primary outcome 11
7.3 Analysis of the secondary outcomes 13

8 REFERENCES 14

APPENDICES 16

Appendix B: Dummy Tables 18

1 ABBREVIATIONS AND GLOSSARY

AE	Adverse Event	PI	Principal Investigator
AR	Adverse Reaction	PIS	Participant Information Sheet
BAFF	B cell activating factor also known as BLyS : B-Lymphocyte Stimulator	PP	Per Protocol
BCDT	B Cell Depletion Therapy	QA	Quality Assurance
BILAG	British Isles Lupus Assessment Group	QC	Quality Control
C3	Complement component 3	R&D	Research and Development
C4	Complement component 4	REC	Research Ethics Committee
CI	Chief Investigator	SAE	Serious Adverse Event
CRF	Case Report Form	SAP	Statistical Analysis Plan
CRP	C-reactive protein	SAR	Serious Adverse Reaction
CTA	Clinical Trial Authorisation	SLE (Lupus)	Systemic lupus erythematosus
CCTU	Comprehensive Clinical Trials Unit	SOC	Standard of care
DNA	deoxyribonucleic acid	SPC	Summary of Product Characteristics
DSUR	Development Safety Update Report	SSA	Site Specific Approval
EU	European Union	SUSAR	Suspected Unexpected Serious Adverse Reaction
ENA	Extractable nuclear antigens	TMF	Trial Master File
GCP	Good Clinical Practice	TMG	Trial Management Group
ICH	International Conference on Harmonisation	TMT	Trial Management Team
IgA	immunoglobulin A	ToR	Terms of Reference
IgG	immunoglobulin G	TSC	Trial Steering Committee
IgM	immunoglobulin M	UCL	University College London
IMP	Investigational Medicinal Product	DMC	Data Monitoring Committee
IRB	Institutional Review Board	C-SSRS	Columbia Suicide Severity Rating Scale
ITT	Intention to Treat	SLEDAI	Systemic Lupus Erythematosus Disease Activity Index
MHRA	Medicines and Healthcare products Regulatory Agency	WCBP	Women of child bearing potential
SDI	SLICC Damage Index		

2 TRIAL SUMMARY & CLINICAL CONTEXT

2.1 Clinical background and rationale

Systemic lupus erythematosus (SLE) is a chronic systemic autoimmune disease with a prevalence of 40 to 200 per 100,000 people, mainly affecting women of child-bearing age. There is a substantial morbidity and mortality associated with lupus, with standardised mortality ratios ranging between 2 and 5 [1]. There is also a lack of novel treatments for patients with severe SLE. Due to the lack of effective alternative therapies many patients require high dose glucocorticoid therapy which is associated with serious adverse effects including increased infections, cataracts, diabetes mellitus and osteoporosis [2]. Both the disease itself and steroid exposure lead to increased rates of cardiovascular disease [3].

A key objective for treatment of severe SLE is disease remission induction the prevention of ‘flare’; (re-)occurrence of lupus symptoms in one or more systems of the body. It is expected that flares will be too rare in this phase II trial for any difference between treatment arms to be reliably detected, so anti-dsDNA antibody levels, which are a sensitive marker of immune system activity associated with lupus flares [4], are the primary outcome instead. Flares are a secondary outcome.

The biologic Rituximab is currently the treatment of choice for refractory cases of SLE where other treatments have not succeeded, though no Randomised Controlled Trials (RCTs) have demonstrated its effectiveness [5]. Previous studies have found that anti-dsDNA levels are increased in patients treated with Rituximab who then flared, indicating two effects of the medication: B cell depletion, which reduces flare risk, but also increasing levels of serum B-cell activating factor/B-lymphocyte stimulator (BAFF/BLyS) in certain patients, which increases flare risk [6]. These opposing effects may explain the lack of significant efficacy found in the previous trials of Rituximab.

There are indications that Belimumab may be an effective addition to Rituximab in this context, as it reduces BAFF levels. Anti-dsDNA is a useful surrogate outcome to evaluate the effect of Belimumab as an adjunct to Rituximab, due to being correlated both with BAFF as well as flare activity.

Another marker of treatment effectiveness is changes in patient’s steroid dose. Patients participating in BEAT-LUPUS will typically be on steroids or both steroids and immunosuppressants at the time of enrolment. During the trial patients may take the steroid prednisolone and one immunosuppressant (either azathioprine, methotrexate, or mycophenolate). In usual care their doctor will reduce their steroid dose if their condition improves and increase it if it deteriorates. Doctors participating in BEAT-LUPUS are asked to reduce their patient’s steroid dose if it is over 10mg/day following administration of Rituximab and Belimumab/placebo. Differences between treatment arms in the extent to which steroid dose is actually reduced, and then maintained at a lower dose, will be partly determined by whether the treating clinician considers that this is safe and tolerable based on clinical symptoms following administration of Belimumab or placebo.

2.2 Trial objectives

The primary objective of BEAT-LUPUS is to compare anti-dsDNA levels 52 weeks after randomisation between a 52-week regime of either Belimumab or placebo amongst patients previously treated with Rituximab. Lupus flares, incidence of adverse events, and changes in dosing of prednisolone, are secondary outcomes. A supportive analysis will seek to examine whether any observed reductions in anti-dsDNA are mediated by changes in the prednisolone dose during follow up.

3 STUDY METHODS

3.1 Design, randomisation, outcomes, and interim stopping rules

BEAT-LUPUS is a UK multicentre, phase II, randomised, double blind, placebo-controlled clinical trial comparing safety and efficacy of a monthly regime of either Belimumab or placebo commencing 4-8 weeks after B cell depletion therapy (Rituximab). The total treatment period (on Belimumab or placebo) will be 52 weeks. There will be an additional follow up appointment at 56 weeks and a pregnancy check at 68 weeks. 56 patients will be recruited and randomised 1:1 to receive either Belimumab or placebo for the 52 week period following completing treatment with Rituximab.

Randomisation will be done using minimisation incorporating a random element to ensure unpredictability in treatment allocations. Factors minimised on include CD19 count at randomisation ($<0.01x10^9/l$ vs $\geq 0.01x10^9/l$) to account for variability in B cell depletion from Rituximab which would affect response; anti dsDNA (positive or negative at first screen); and whether patients have active renal disease at screening.

For the primary analysis an ANCOVA model (see Section 6 below) will be done which will examine the treatment difference at 52 weeks and test for superiority. The measurement taken closest to the 52 week point of follow up will be used, with measurements taken up to 2 months before or after the 52 week point being eligible for inclusion in the analysis.

No formal interim analyses will be done. An Independent Data Monitoring Committee (IDMC) will meet annually to review safety and efficacy data, and they may recommend stopping the trial if they judge the results are likely to convince a broad range of clinicians that one arm is clearly indicated or contraindicated.

3.2 Sample size calculation

Sample size calculations were performed using STATA 13. The calculation assumed that anti dsDNA binding antibody levels are log normally distributed, that an ANCOVA analysis model would be used to evaluate difference in mean log anti-dsDNA between treatment arms at 52 weeks [7], and made additional adjustment for expected losses to follow up.

The Standard Deviation of anti dsDNA and the correlation structure assumed were based on two sets of data: (a) the study of 35 participants by Carter et al [6] (b) data provided by David Isenberg for 67 participants before and 6 months after B cell depletion therapy.

Table 1: Standard deviations and correlations for log anti dsDNA

Dataset	Time point	Log mean	Log sd	Correlation
**Isenberg (n=67)**	**Baseline**	6.036	1.393	
	**Follow up**	5.189	1.500	0.612
**Carter (n=35)**	**Baseline**	5.091	1.676	
	**Follow up**	5.225	1.781	0.527

Based on the above data, the standard deviation of final anti dsDNA measurements was estimated to be 1.7, and the correlation between baseline and final measurements was estimated to be 0.55. 22 evaluable participants per group would be sufficient to detect a difference of 1.2 in log anti dsDNA at 5% significance with 80% power. We assume that 20% of participants will fail to attend the 12 month follow-up visit, so aim to recruit 28 participants per group.

The study's power to detect a difference of 1.2 in log anti-dsDNA is equivalent to being able to detect a difference of 232% between arms (equivalent to multiplying by exp(1.2)). To place this in context, Carter *et al* found the log difference between participants who did and did not have a flare was 1.928, corresponding to a 588% increase in anti dsDNA amongst participants who flared [6].

4 STATISTICAL PRINCIPLES

2-sided p-values and 95% confidence intervals will be reported for all statistical tests. There is only one pre-specified primary analysis, which will use a p-value threshold of 5% to reject the null to ensure that the probability of a type I error does not exceed 5%. Log anti-dsDNA will be used to account for skewness in anti-dsDNA measurements.

Adherence to the protocol requires that the patient (a) takes the treatment they were randomised to for 52 weeks (b) does not exceed pre-specified doses of concomitant medications at enrolment and that these do not increase during follow up. Patients are encouraged to continue providing follow-up measurements, even if they stop adhering to the protocol before 52 weeks.

The percentages of patients who (i) fully adhere to the protocol (ii) do not adhere but do provide a 52 week measurement, will be reported. The primary analysis will be intention-to-treat; all patients who provide a 52-week measurement will be included regardless of adherence to the protocol. Secondary analyses will be done which will only include patients who adhered to the protocol.

5 TRIAL POPULATION

The full eligibility criteria for enrolment into BEAT-LUPUS are listed in the trial protocol and Appendix 1 of this document.

Counts of patients screened but not enrolled in the trial and reason for exclusion will be reported, and recruitment to the trial will be presented by centre and calendar month. The number of patients who withdraw or are unwilling to continue in follow-up will be reported by last follow-up visit attended and treatment arm. Reasons for patient withdrawals will be tabulated by treatment arm. The full throughput of patients from screening to analysis will be summarised in a CONSORT flowchart [8].

Baseline characteristics of patients in BEAT-LUPUS will be summarised by treatment arm. Characteristics described will include screening anti-dsDNA, CD19 count, presence of renal disease, age, and sex. Characteristics at randomisation will also be reported: biomarker levels including anti-dsDNA, CD19 levels, and current doses of concomitant medications. Characteristics will be summarised using means and standard deviations for (approximately) normally distributed continuous variables, geometric means and 95% confidence intervals for (approximately) log normally distributed continuous variables, medians and interquartile ranges for non-normally distributed variables, and frequencies and percentages for categorical variables.

Figure 1. Participant Timeline

Procedure/ Activity	Screening	Wk -8	Wk -1	Day 0	Wk2	Wk4	Wk8	Wk 12	Wk16	Wk20	Wk24	Wk28	Wk32	Wk36	Wk40	Wk44	Wk48	Wk52	Wk 56	**Flare/ Withdrawal**
Rituximab Infusion		x																		
Randomisation				x																
Trial infusion				x	x	x	x	x	x	x	x	x	x	x	x	x	x	x		
Review of AEs/SAEs				x	x	x	x	x	x	x	x	x	x	x	x	x	x	x	x	x
Anti-dsDNA	x		x					x			x							x		x
CD19	x		x					x			x							x	x	x
Complement C3, C4	x		x					x			x							x	x	x
Immunoglobulins IgG, IgA, IgM	x		x					x			x							x	x	x
SLEDAI 2000 disease activity	x		x					x			x							x	x	x
BILAG 2004 lupus activity	x			x		x	x	x	x	x	x	x	x	x	x	x	x	x	x	x
SGADA subjective disease activity	x			x		x	x	x	x	x	x	x	x	x	x	x	x	x	x	x
LUPUS QoL	x			x				x			x			x				x	x	x
SF-36 health state	x			x				x			x			x				x	x	x
C-SSRS suicide severity	x			x	x	x	x	x	x	x	x	x	x	x	x	x	x	x	x	x
HAQ disability	x							x			x							x	x	x
SLICC damage index	x							x			x							x	x	x
EQ5D health state	x			x				x			x			x				x	x	x

6 TRIAL OUTCOMES

6.1 Primary and secondary outcomes

The primary outcome measure is log anti dsDNA-antibody levels at 52 weeks. Secondary outcomes are:

1. Log anti-dsDNA antibody levels at 12 and 24 weeks.
2. Proportion of participants with any adverse events and proportion with any serious adverse events
3. Proportion of participants with any infections
4. Proportion of participants with any severe flare (severe flare: a BILAG A score due to items which are “new” or “worse”; or, in the renal or haematological systems, an A score due to items which didn’t result in an A score last month) by 24 and 52 weeks, and time to severe flare
5. Proportion of participants with any severe flare or a moderate flare (moderate flare: 2 BILAG B scores due to items which are either “new” or “worse”; or, in the renal or haematological systems, B scores due to items which didn’t result in a B score last month) by 24 and 52 weeks, and time to severe or moderate flare
6. Proportion of participants with any severe flare, moderate flare, or mild flare (mild flare: A single B score due to items which are “new” or “worse”; or, for the renal or haematological systems, a B score due to items which didn’t result in a B score last month) by 24 and 52 weeks, and time to severe, moderate or mild flare
7. Proportion of participants with any severe or moderate accompanied by an increase in concomitant lupus medication - glucocorticoids, Mycophenolate, Azathioprine, or Methotrexate by 24 and 52 weeks, and time to any flare
8. SLEDAI 2000 at 52 weeks
9. The SLICC/ACR Damage Index (SDI) at 52 weeks
10. Visual Analogue Scale (VAS) Subject Global Assessment of Disease Activity (SGADA) at 52 weeks
11. C3 at 52 weeks
12. Immunoglobulin levels at 52 weeks
13. Cumulative steroid and immunosuppressant doses during treatment from randomisation to 52 weeks
14. Proportion of participants successfully reducing their steroid dose at the time of randomisation: decreasing their steroid dose by 50% without flaring; or if below 10mg/day at randomisation, reducing steroid dose to 5mg/day; or who discontinue steroids with stable disease.
15. Proportion of participants with a prednisolone dose <= 7.5 mg/day at both weeks 48 and 52.
16. Lupus Quality of Life (Lupus QoL), SF-36 and EQ5D at 52 weeks
17. C-SSRS (Columbia suicide severity rating scale) to assess suicide risk at 52 weeks
18. HAQ at 52 weeks

6.2 Scoring and description of derived outcome measures

British Isles Lupus Assessment Group (BILAG-2004) Index

The BILAG-2004 questionnaire comprises 97 questions on lupus activity in the past four weeks compared to the previous four weeks, for each of nine constitutional systems of the body [9]. Individual items can be scored either on a 0-4 scale from 'Not present' (0) to 'new' (4), or as numerical measurements. An algorithm is then applied to determine an overall 'flare' score for each system; A=severe disease activity, B=moderate disease activity, C=mild disease, D=inactive disease but previously affected, and E=system never involved. Additional criteria are applied to identify A and B scores which are new manifestations of the disease. Then the scores are used to determine if an overall disease flare occurred. 'Severe' flare occurs if there is at least one A score due to items which are "new" or "worse" on the BILAG questionnaire; or, in the renal and haematological systems, due to questionnaire items which last month did not result in an A score (i.e. which were less severe). A 'moderate' flare occurs if at least two new B scores occur which are due to items which are "new" or "worse", or, in the renal and haematological systems, due to items which last month did not result in a B score (i.e. were less severe). A 'mild' flare occurs if there is only one B score which meets these conditions.

The subset of BILAG flares which are also accompanied by an increase in one of the medications used to control the disease can also be evaluated. This allows evaluation of only those flares which were severe enough in the clinician's judgement to modify the treatment regime.

The Systemic Lupus Erythematosus Disease Activity Index-2000 (SLEDAI)

The SLEDAI Responder Index determines improvement in Lupus activity based on 24 items in 9 organs in the previous 30 days [10]. The scores from the different systems are weighted in proportion to their hazard (i.e. central nervous system items are weighted twice that of joint pain and kidney disease) and combined into one final score from 0-105.

Patient global assessments of lupus activity on a 10cm visual analogue scale (VAS)

This is a BEAT-LUPUS specific measure of disease activity developed for this trial. Patients are presented with a line labelled 0-10, and point to the number on the line which best matches their own assessment of lupus activity in terms of lupus associated symptoms in the past 4 weeks (0 – not active at all, 10 – extremely active).

LUPUSQoL

The LUPUSQol measure is a lupus-specific health-related quality of life measure [11]. It comprises 34 questions which each ask about effects of lupus on day-to-day physical and emotional health, body image, pain, planning, fatigue, intimate relationship, and burden to others. Patients answer each question on a scale of (1) "All of the time" to (5) "Never". Average scores for each domain are mapped to a 0-100 score. So long as 50% of data items for a domain are completed a 0-100 score will be calculated, in line with guidance from the

authors of the questionnaire [11]. The mean score across domains is then calculated as the average of the domain-specific scores.

The Short Form (36) Health Survey (SF-36)

The SF-36 is a survey of patient health in eight sections: vitality, physical functioning, bodily pain, general health perceptions, physical role functioning, emotional role function, social role functioning, and mental health [12]. Each section has a score which is a weighted sum of the questions in that section, directly transformed into a 0-100 score, with lower scores indicating more disability. Unanswered questions are excluded; the average for all items on the scale that the respondent answered is used instead. A standardised composite score of health is then generated from each of the eight scores.

Columbia Suicide severity Rating Scale (C-SSRS)

The C-SSRS questionnaire provides summary measures of suicidal ideation and behaviour. These are strongly associated with risk of individual's completing suicide [13]. The ideation and behaviour sections can be scored separately and also combined into one summary score [14]. Ideation is scored at each visit from (1) "Wish to be dead" to (5) "Active suicidal ideation with specific plan and intent"; behaviour is scored from (6) "Preparatory acts or behaviour" to (10) "Completed Suicide". Imputation of missing values is not done; if any data is missing for a domain its score is not calculated.

The Stanford HAQ 20-item Disability Scale (HAQ)

This is a questionnaire which summarises patient disability based on extent of difficulty within 8 domains; dressing and grooming, arising, eating, walking, hygiene, reach, grip, and activities [15]. The total score is the mean score of the eight category scores. If more than two of the categories are missing the score is not calculated. If only one category is missing, the mean of the other seven category scores is used as the total score.

The Systemic Lupus International Collaborating Clinics / American College of Rheumatology (SLICC/ACR) damage Index for Systemic Lupus Erythematosus

The SLICC/ACR Damage Index (SDI) provides a measure of accumulated damage in the body since the onset of Lupus [16]. It is a summary score based on damage across 12 different organ systems. For each system, a variety of different possible types of damage or disease are listed; the presence of one results in a score of 1 or 2. The summary score for the whole body is the sum of all the individual scores.

EQ-5D-5L

The EQ-5D-5L assesses current health state across five dimensions: mobility, self-care, usual activities, pain/discomfort, and anxiety/depression with five levels (each scored 1 - 5, with higher scores indicating worse health state) [17]. EQ-5D dimension scores will be converted to index scores using UK population values. EQ-5D index scores range from -1=worse than death, and then 0=worst to 1=best health state. The EQ-5D additionally includes a visual analogue scale (EQ VAS), which allows patients to record their overall current health status on a scale ranging from 0=worst to 100=best health state.

If any dimension score is missing the EQ-5D index score will be set to missing. If the entirety of one component of the questionnaire (dimension score or VAS) has not been completed the associated component score will be set to missing. If the entire questionnaire has not been completed, both the EQ-5D index score and EQ-5D VAS at that visit will be set missing.

7 STATISTICAL ANALYSIS

The results of the analyses will be reported following the principle of the ICH E3 guidelines on the Structure and Content of Clinical Study Reports. All analyses will be performed using STATA [18]. (8). In addition the primary analysis of the primary outcome, mediation and other secondary analyses of the primary outcome will also be done, and results for the primary outcomes will also be presented by levels of the stratifying variables adjusted for in the primary analysis, as an exploratory subgroup analysis. For all analyses done using linear regression models, diagnostic checks will be done using residual plots and the data will be transformed and re-analysed if necessary.

7.1 Primary analysis of the primary outcome

A linear regression ANCOVA model will be fitted to evaluate difference in 52-week anti-dsDNA between treatment arms, adjusting for CD19 count at randomisation ($<0.01x10^9$/l vs $\geq 0.01x10^9$/l, previous renal involvement (yes/no) at screening, anti-dsDNA levels at screening (positive/negative) and also anti-dsDNA levels measured at randomisation. The model will be specified as follows, where $Y_{i,j}$ is the anti-dsDNA of patient j at time i:

$$\log(Y_{52,j}) = \beta_0 + \beta_1(\text{treatment}_j) + \beta_2(\text{CD19}_j) + \beta_3(Y_{0,j}) + \beta_4(\text{renal}_j) + \beta_4(\text{screenDNA}_j) + \varepsilon_j$$

Where treatment_j = 1 if Belimumab and 0 if placebo, and ε_{ij} is a normal error distribution. The primary outcome will be estimated by $\exp(\beta_1)$ as the difference in anti-dsDNA amongst patients randomised to Belimumab compared to the placebo group at 52 weeks, expressed as a percentage of the average in the placebo group at 52 weeks.

7.2 Supportive analyses of the primary outcome

Analysis of log anti-dsDNA at 12 and 24 weeks

The model structure in 7.1 will also be repeated with the outcome changed to log anti-dsDNA at 12 and 24 weeks to evaluate differences between treatment arms at these time points. These analyses will be done on the intention-to-treat basis, the same as the primary analysis.

Per-protocol repeated measures analysis of anti-dsDNA at 52 weeks

Repeated measures linear regression will be used to analyse the difference between arms in anti-dsDNA using the randomisation and all follow up measurements in the same model, and excluding measurements made after the point at which the patients stops adhering to the protocol (either after the time the patient stops taking their randomised treatment or

after increasing the dose of one of the allowed concomitant medications). This model will estimate the mechanistic effect of Belimumab on anti-dsDNA.

In the model Patient ID will be included as a random effect to account for correlation between measurements on the same patient at different points of follow up. The model for anti-dsDNA at 52 weeks, where y_{ij} is the anti-dsDNA of patient j at time i, is

$$y_{ij} = \beta_{0j} + \beta_1(\text{time}_i) + \beta_2(\text{time}_i{*}\text{treatment}_j) + \beta_3(\text{CD19}_j) + \beta_4(\text{renal}_j)$$

Where, $\beta_{0j} = \beta_0 + u_{0j} + \varepsilon_{ij}$

And, $u_{0j} \sim N(0, \sigma_{u0}^2)$
$\varepsilon_{ij} \sim N(0, \sigma^2)$

And, $\text{treatment}_j = 1$ if Belimumab and 0 if placebo.

The average treatment difference at 52 weeks will be estimated by β_2*52. Log-transformation of anti-dsDNA or fractional polynomials for the effect of time will be considered if plots of residuals or likelihood ratio tests indicate these will improve the model fit.

Mediation analysis of the effect of prednisolone on anti-dsDNA at 52 weeks
If material differences (P<0.1) between treatment arms are found in the cumulative prednisolone dose between randomisation and 52 weeks, an exploratory causal mediation analysis will be done to evaluate the extent to which this may mediate any effect of allocation to Belimumab on anti-dsDNA at 52 weeks [19]. The direct effect of Belimumab (i.e. the effect of taking Belimumab instead of placebo, had the cumulative steroid dose been the same in both conditions) and the average causal mediation effect (i.e. the effect of the cumulative steroid dose patients would have taken on Belimumab instead of the dose they would have taken on placebo, had they actually taken Belimumab in both conditions) will be estimated using the STATA *mediation* package [20].

Sensitivity analysis for informative loss to follow up
If over 10% of patients fail to provide a 52-week anti-dsDNA measurement, a sensitivity analysis will be done using multiple imputation to evaluate whether the primary analysis and the repeated-measures per protocol analysis are biased by missing data. Where missing, anti-dsDNA measurements will be imputed for patients missing them using all variables in the primary analysis model and data on concomitant medications, flares and time to flare, all other available anti-dsDNA measurements from other scheduled visits, and any anti-dsDNA measurements taken at point of flare/withdrawal. A number of imputation datasets sufficient to give a power reduction of <1% compared to using n=100 will be produced [20]; the analysis models will be run on each of these datasets; and estimates and confidence intervals will be combined using Rubin's rules [21]. The concordance of results between the non-imputation (complete case) and imputation models will be assessed.

7.3 Analysis of the secondary outcomes

The percentage of patients with the following characteristics will be compared between treatment arms using Fisher's exact test:

I. BILAG severe flare
II. BILAG severe or moderate flare
III. BILAG severe, moderate or mild flare
IV. BILAG severe or moderate flare which was accompanied by an increase in one or more concomitant medication
V. Any serious adverse event
VI. Any infection
VII. Any adverse events
VIII. Completed 52 weeks follow up
IX. Completed 52 weeks treatment

For each of SLEDAI, SLICC, VAS, C3, Immunoglobulin levels, and LupusQol, SF-36, and EQ-5D-DL, assessments at 52 weeks will be compared between arms using linear regression models which includes the stratifying variables and the value of the variable at screening (for the HAQ and SLICC) or randomization (for all others). Time to disease flare will be visually displayed using Kaplan-Meier curves, and difference between arms in hazard of flare will be tested using Cox models which include the stratifying variables. For the BILAG flare scores, an ordinal logistic regression model will also be fitted to compare maximum disease flare severity experienced during follow up (severe, moderate, mild, or no flare), also adjusted for the stratifying variables.

Differences between the arms in the following steroid dose summary dose will be compared:

i) The cumulative steroid dose from randomisation to 52 weeks using a two sample t test;
ii) Proportion of participants successfully reducing their steroid dose, using Fisher's exact test.
iii) Proportion of patients taking <=7.5mg of prednisolone at weeks 48 and 52.

The following quantities taken from the C-SSRS will be compared between treatment arms:

i) Average C-SSRS score at 52 weeks.
ii) Percentage of patients with a C-SSRS score which increased to >5 at any point of follow up.

For the questionnaires completed at each follow up visit (BILAG, VAS, and C-SSRS), if the questionnaire is not completed at one visit, the result from the previous month will be carried forward for one month only (unless it is missing due to withdrawal/flare since the previous visit, in which case data captured at that point will be used).

8 REFERENCES

1. Thomas, G., et al., *Mortality associated with systemic lupus erythematosus in France assessed by multiple-cause-of-death analysis.* Arthritis Rheumatol, 2014. **66**(9): p. 2503-11.
2. Ruiz-Irastorza, G., A. Danza, and M. Khamashta, *Glucocorticoid use and abuse in SLE.* Rheumatology (Oxford), 2012. **51**(7): p. 1145-53.
3. Ahmad, Y., et al., *Subclinical atherosclerosis in systemic lupus erythematosus (SLE): the relative contribution of classic risk factors and the lupus phenotype.* Rheumatology (Oxford), 2007. **46**(6): p. 983-8.
4. Lazarus, M.N., et al., *B-cell numbers and phenotype at clinical relapse following rituximab therapy differ in SLE patients according to anti-dsDNA antibody levels.* Rheumatology (Oxford, England), 2012. **51**(7): p. 1208-1215.
5. Favas, C. and D.A. Isenberg, *B-cell-depletion therapy in SLE--what are the current prospects for its acceptance?* Nat Rev Rheumatol, 2009. **5**(12): p. 711-6.
6. Carter, L.M., D.A. Isenberg, and M.R. Ehrenstein, *Elevated serum BAFF levels are associated with rising anti-double-stranded DNA antibody levels and disease flare following B cell depletion therapy in systemic lupus erythematosus.* Arthritis Rheum, 2013. **65**(10): p. 2672-9.
7. Borm, G.F., J. Fransen, and W.A. Lemmens, *A simple sample size formula for analysis of covariance in randomized clinical trials.* J Clin Epidemiol, 2007. **60**(12): p. 1234-8.
8. Schulz, K.F., D.G. Altman, and D. Moher, *CONSORT 2010 Statement: updated guidelines for reporting parallel group randomised trials.* BMJ, 2010. **340**.
9. Gordon, C., et al., *Definition and treatment of lupus flares measured by the BILAG index.* Rheumatology, 2003. **42**(11): p. 1372-1379.
10. Bombardier, C., et al., *Derivation of the sledai. A disease activity index for lupus patients.* Arthritis & Rheumatism, 1992. **35**(6): p. 630-640.
11. McElhone, K., et al., *Development and validation of a disease-specific health-related quality of life measure, the LupusQol, for adults with systemic lupus erythematosus.* Arthritis Care & Research, 2007. **57**(6): p. 972-979.
12. *36-Item Short Form Survey (SF-36)*. RAND Corporation.
13. Kelly Posner, et al., *The Columbia–Suicide Severity Rating Scale: Initial Validity and Internal Consistency Findings From Three Multisite Studies With Adolescents and Adults.* American Journal of Psychiatry, 2011. **168**(12): p. 1266-1277.
14. *Columbia-Suicide Severity Rating Scale Scoring and Data Analysis Guide*. 2013, The Columbia Lighthouse Project.
15. Bruce, B. and J.F. Fries, *The Stanford Health Assessment Questionnaire: dimensions and practical applications.* Health and quality of life outcomes, 2003. **1**: p. 20-20.
16. Gladman, D., et al., *The development and initial validation of the systemic lupus international collaborating clinics/American college of rheumatology damage index for systemic lupus erythematosus.* Arthritis & Rheumatism, 1996. **39**(3): p. 363-369.
17. Herdman, M., et al., *Development and preliminary testing of the new five-level version of EQ-5D (EQ-5D-5L).* Quality of Life Research, 2011. **20**(10): p. 1727-1736.
18. *STATA: Release 15. Statistical Software.* . College Station, TX: StataCorp LLC.

19. Fritz, M.S., A.B. Taylor, and D.P. Mackinnon, *Explanation of Two Anomalous Results in Statistical Mediation Analysis.* Multivariate behavioral research, 2012. **47**(1): p. 61-87.
20. Graham, J.W., A.E. Olchowski, and T.D. Gilreath, *How many imputations are really needed? Some practical clarifications of multiple imputation theory.* Prev Sci, 2007. **8**(3): p. 206-13.
21. Carpenter, J. and M. Kenward, *Multiple imputation and its application*. 2013, Chichester: Wiley.

APPENDICES

Appendix A: Inclusion and exclusion criteria for BEAT-LUPUS

Inclusion criteria:

1. Aged between 18 and 75 years
2. Participants with 4 or more criteria for SLE according to the American College of Rheumatology (ACR) 1997 criteria or SLICC 2012 criteria or biopsy proven lupus nephritis with one additional supportive test on at least two occasions (positive ANA, anti-dsDNA antibodies or anti-Sm antibodies)
3. History of anti-dsDNA antibodies detectable at least once in the past 5 years prior to screening the participant on the study protocol (ELISA test is preferable for Anti dsDNA antibody testing).
4. Participants are due to be treated with the first infusion of this cycle of B cell depletion therapy (Rituximab) 4-8 weeks before randomisation (Day 0, see participant timeline). Previous use of Rituximab is allowed prior to this cycle.
5. No contraindications to the use of Belimumab.
6. Ability to provide informed consent

Exclusion criteria:

1. Severe “critical” SLE flare defined as BILAG A flare in CNS system or any SLE manifestation requiring more immunosuppression than allowed within the protocol in the physician's opinion
2. Pregnancy and/or Breast Feeding participants
3. At risk of pregnancy and unwilling to use an acceptable form of birth control contraception (see section 6.3.1.4)
4. Prior use of Belimumab, Atacicept or any biologic therapy (except Rituximab, but no other B cell depleting therapies)
5. Participation in any other interventional trial within the last 6 months
6. eGFR <30mls/min at screening
7. Active infections, including but not limited to:

i. Current or past infection with hepatitis B or C as defined by:

A. Hepatitis B surface antigen positive

B. Hepatitis B surface antibody positive and hepatitis B core antibody positive

C. Hepatitis C antibody positive

ii. Historically positive HIV test or test positive at screening for HIV

iii. Active TB.

8. Infection history:

i. Currently on any suppressive therapy for a chronic infection (such as tuberculosis, pneumocystis, cytomegalovirus, herpes simplex virus, herpes zoster and atypical mycobacteria)

ii. Hospitalization for treatment of infection within 60 days of Day 0

iii. Use of parenteral (IV or IM) antibiotics (antibacterials, antivirals, anti-fungals, or anti-parasitic agents) within 30 days of Day 0

9. Receipt of a live-attenuated vaccine within 3 months of Day 0 (see participant timeline)
10. In the investigator's opinion, participants that are at high risk for infection (including but not limited to in dwelling catheter, dysphagia with aspiration, decubitus ulcer, history of prior aspiration pneumonia or recurrent severe urinary tract infection)
11. IgG levels below 4.0 g/L, IgA level < 10 mg/dL (IgG and IgA test must be performed no more than 10 days before study drug started for the second inclusion/exclusion criteria assessment at Day 0)
12. Primary immunodeficiency
13. History of malignant neoplasm within the last 5 years
14. History of cervical dysplasia CIN Grade III cervical high risk human papillomavirus or abnormal cervical cytology other than abnormal squamous cells of undetermined significance (ASCUS) within the past 3 years. The participant will be eligible after the condition has resolved (e.g., follow-up HPV test is negative or cervical abnormality has been effectively treated >1 year ago)
15. Severe, progressive, or uncontrolled renal, hepatic, haematological, gastrointestinal, pulmonary, cardiac, or neurological disease or, in the investigator's opinion, any other concomitant medical condition or significant abnormal laboratory value that places the participant at risk by participating in this study with the exception of diseases or conditions related to active SLE.
16. Comorbidities, not lupus related currently requiring systemic corticosteroid therapy.
17. Evidence of serious suicide risk including any history of suicidal behaviour in the last 6 months and/or any suicidal ideation in the last 2 months or who in the investigator's judgement, pose a significant risk.
18. History of an anaphylactic reaction to parenteral administration of contrast agents, human or murine proteins or monoclonal antibodies.
19. Current drug or alcohol abuse or dependence, or a history of drug or alcohol abuse or dependence within 364 days prior to Day 0
20. White blood cells (WBC) <1.5 x 109/L, Neutrophils <1 x 109/L measured up to 10 days before Day 0 (study drug commenced)
21. A history of major organ transplant or hematopoietic stem/cell/marrow transport or renal transplant.

Appendix B: Dummy Tables

Table 1: Patient characteristics at screening (or at randomisation, if specified)

Characteristic		Belimumab<br>n=	Placebo<br>n=	Total<br>n=
Age (years)	mean(sd)			
Female	n(%)			
Weight (kg)	mean(sd)			
Active renal disease	n(%)			
CD19≥$0.01x10^9$/L	n(%)			
BILAG A or B<br>Constitutional<br>Mucocutaneous<br>Neuropsychiatric<br>Musculoskeletal<br>Cardiorespiratory<br>Gastrointestinal<br>Ophthalmic<br>Renal<br>Haematological	n(%)			
SLEDAI 2000<br>Score ≥10<br>*Organ involvement*<br>CNS<br>Serosal<br>Haematological<br>Constitutional<br>Immunological<br>Musculoskeletal<br>Dermal<br>Renal Vascular	mean(sd)<br>n(%)<br><br>n(%)			
SLICC damage index	mean(sd)			
Subjective GADA	mean(sd)			
Lupus QoL<br>Physical Health<br>Emotional Health<br>Body Image<br>Pain<br>Planning<br>Fatigue<br>Intimate relationships<br>Burden to others	mean(sd)			

SF36<br>Vitality<br>Physical functioning<br>Bodily pain<br>General health<br>Physical role<br>Emotional role<br>Social role<br>Mental health	mean(sd)			
C-SSRS<br>Ideation<br>Behaviour	mean(sd)			
HAQ<br>Total<br>Dressing & Grooming<br>Arising<br>Eating<br>Walking<br>Hygiene<br>Reach<br>Grip<br>Activities	mean(sd)			
SDI	mean(sd)			
EQ-5D-5L	mean(sd)			
VAS	mean(sd)			
Medications at randomisation<br>Daily prednisolone use<br>≥7.5mg/day<br>Any immunosuppressant use<br>Mycophenolate mofetil<br>Azathioprine<br>Methotrexate<br>Antimalarial use	 Mean(sd)<br>n(%)<br><br><br>n(%)			
Biomarkers at randomisation<br>Anti-dsDNA antibodies IU/ml<br>C3 g/L<br>C4 g/L<br>IgG g/L<br>IgA g/L<br>IgM g/L	mean(sd)			
B cell subsets at randomisation<br>CD19 $x10^9$/L	 mean(sd)			

Table 2: Primary outcome, disease flares and adverse events

Outcome		Belimumab<br>n=	Placebo<br>n=	Effect# (95% CI)	p-value
**Protocol compliance & concomitant medications**					
Completed 52 wks follow up	n(%)				
Completed 52 wks treatment	n(%)				
Cumulative steroid dose from randomisation to 52wks	mean(95%CI)				
Proportion of patients successfully reducing steroid dose by 50% or to 5mg/day without flaring	n(%)				
Proportion of patients taking <=7.5 mg/day prednisolone at both weeks 48 and 52	n(%)				
**Anti-dsDNA**					
**Anti-dsDNA antibodies IU/ml at 52wks****	Geometric mean (95%CI)				
Anti-dsDNA antibodies IU/ml at 24wks	Geometric mean(95%CI)				
Anti-dsDNA antibodies IU/ml at 12wks	Geometric mean(95%CI)				
Per-protocol repeated measures analysis estimates of Anti-dsDNA at 52wks	mean(95%CI)				
**Disease flares**					
Proportion with severe flare by 52wks	n(%)				
Proportion with severe flare by 24wks	n(%)				
Time to severe flare, wks	median(95%CI)				
Proportion with severe/moderate flare by 52wks	n(%)				
Proportion with severe/moderate flare by 24wks	n(%)				
Time to severe/moderate flare, wks	median(95%CI)				
Proportion with severe/moderate/mild flare by 52wks	n(%)				
Proportion with severe/moderate/mild flare by 24wks	n(%)				
Time to severe/moderate/mild flare, wks	median(95%CI)				
Proportion with severe or moderate flare followed by increase in a concomitant medication by 52wks	n(%)				
Proportion with severe or moderate flare followed by increase in a concomitant medication by 24wks	n(%)				
Time to severe or moderate flare followed by increase in a concomitant medication	median(95%CI)				

Odds ratio for having flare of greater severity (severe, moderate, mild, compared to no flare) from ordered logit model					
**Adverse events**					
Proportion with any SAEs by 52wks	n(%)				
Proportion with any infections by 52wks	n(%)				
Proportion with any adverse events by 52wks	n(%)				

Unless stated otherwise the intention to treat population is used, and effect estimates for continuous variables are from ANCOVA linear regression models which adjust for renal activity, anti-dsDNA at screening, and CD19 count and anti-dsDNA at randomisation. Those covariates are also adjusted for in the Cox regression models for time to flare, and ordered logit model for odds ratio for flare of greater severity. For anti-dsDNA only, the log(anti-dsDNA) is modelled and the model effect estimate is the percentage difference in anti-dsDNA between arms. Fisher's exact is used for comparison of proportions.

** Primary analysis of primary outcome

Table 4: Secondary outcomes, disease activity, quality of life, and biomarkers at 52 weeks

**Outcome**		**Belimumab** n=	**Placebo** n=	**Effect#** (95% CI)	**p-value**
SLEDAI 2000 score at 52wks	mean(95%CI)				
SLICC Damage Index at 52 wks	mean(95%CI)				
**Patient-reported outcomes**					
Subjective GADA at 52wks	mean(95%CI)				
HAQ<br>Total<br>Dressing & Grooming<br>Arising<br>Eating<br>Walking<br>Hygiene<br>Reach<br>Grip<br>Activities	mean(sd)				
Lupus QoL at 52wks<br>Total<br>Physical Health<br>Emotional Health<br>Body Image<br>Pain<br>Planning<br>Fatigue<br>Intimate relationships<br>Burden to others	mean(sd)				

SF36<br>Vitality<br>Physical functioning<br>Bodily pain<br>General health<br>Physical role<br>Emotional role<br>Social role<br>Mental health	mean(sd)				
Average EQ-5D-5L from randomisation to 52wks	mean(95%CI)				
C-SSRS at 52wks	mean(95%CI)				
C-SSRS increase to >5 at any follow up visit	n(%)				
**Biomarkers**					
C3 at 52wks g/L	mean(95%CI)				
IgG at 52wks g/L	mean(95%CI)				
IgA at 52wks g/L	mean(95%CI)				
IgM at 52wks g/l	mean(95%CI)				
CD19 at 52wks x10^9/L	mean(95%CI)				
[#] Unless stated otherwise the intention to treat population is used, and effect estimates for continuous variables are from ANCOVA linear regression models which adjust for renal activity, anti-dsDNA, and CD19 count at randomisation. Fisher's exact is used for comparison of proportions.					
