# Supplementary material for: Safety and efficacy of belimumab after B cell depletion therapy in systemic LUPUS erythematosus (BEAT-LUPUS) trial: statistical analysis plan
Source: Trials. 2020 Jul 16;21:652. doi: 10.1186/s13063-020-04391-2 (PMC7364494; doi:10.1186/s13063-020-04391-2)
Supplement: Supplementary file 3 — Additional file 3. Guidelines for the Content of Statistical Analysis Plans in Clinical Trials Checklist (as specified in Gamble et al, JAMA, 2017, and recommended by the EQUATOR Network). [file 13063_2020_4391_MOESM3_ESM.docx]

**Additional file 3:** Guidelines for the Content of Statistical Analysis Plans in Clinical Trials Checklist (as specified in Gamble et al, JAMA, 2017, and recommended by the EQUATOR Network)
